# Hybrid state analysis with improved firefly optimized linear congestion models of WSNs for DDOS & CRA attacks

**DOI:** 10.7717/peerj-cs.845

**Published:** 2022-01-27

**Authors:** K Abdul Basith, T.N. Shankar

**Affiliations:** Department of Computer Science and Engineering, Koneru Lakshmaiah Education Foundation, Vaddeswaram, Guntur, Andhra Pradesh, India

**Keywords:** ACO, Clustering, DDOS, FIREFLY, FUZZY Logic, Genetic Algorithms, LCM, LEACH, PDOS, WSN

## Abstract

A decentralized form represents a wireless network that facilitates the computers to direct communication without any router. The mobility of individual nodes is necessary within the restricted radio spectrum where contact is often possible on an Adhoc basis. The routing protocol must face the critical situation in these networks forwarding exploration between communicating nodes may create the latency problem in the future. The assault is one of the issues has direct impact network efficiency by disseminating false messages or altering routing detail. Hence, an enhanced routing approach proposes to defend against such challenges. The efficiency of the designated model of wireless devices relies on various output parameters to ensure the requirements. The high energy efficient algorithms: LEACH with FUZZY LOGIC, GENETIC, and FIREFLY are the most effective in optimizing scenarios. The firefly algorithm applies in a model of hybrid state logic with energy parameters: data percentage, transmission rate, and real-time application where the architecture methodology needs to incorporate the design requirements for the attacks within the specified network environment, which can affect energy and packet distribution under various system parametric circumstances. These representations can determine with the statistical linear congestion model in a wireless sensor network mixed state environment.

## Introduction

Wireless networks can be susceptible to security threats. Interference on the transmission channel is less complicated than on wired networks, and the scrambling frequency bands have launched denial of service attacks (Fang et al., 2016). Based on a wireless model with an ADHOC case study, the design generates a set of failures (Dong, Abbas & Jain, 2019. Implementing the novel scenarios on the nodes is vulnerable to DOS attacks (Chen & Kuo, 2019; Gope, Lee & Quek, 2017). The network security model considers the proposed scheme’s different methods that recognize various attacks and congestion problems. A network must deliver the packets cooperatively using the available tools (Ashfaq, Ali Safdar & Ur-Rehman, 2018). The DDoS vulnerability is one of the most dangerous attacks to recognize on an ad hoc network. The information transfer on the network model provides DDOS information on the current session from source to destination. Affected traffic consumes the network’s bandwidth or the computing resources of the target host, resulting in the rejection of legitimate requests. The energy changes in the design architecture for the network based on the Adhoc model must have provision for the bandwidth features of the different packets, and the service features can attain the traffic congestion and attack vulnerability at every time interval. The only viable alternative is to build a defense device capable of detecting and responding to an attack by reducing excessive traffic (Chen & Kuo, 2019).

In recent times, information technology has flourished in all dimensions. With WSN, an efficient medium of data exchange is to provide different features irrespective of place and time, which is not possible with the interference of human beings. With limited energy sources, a large number of sensors serve as nodes (Imen & Ahlem, 2021). Thus, the WSN faces numerous problems concerning energy distribution (Razzaque & Dobson, 2014). The utilization of power according to the amount of consumption among several components limits the lifetime of the energy sources Doncel, 2021. Thus, it is essential to preserve the energy to maintain a balance in terms of power distribution. Data routing and cluster-head selection are the two significant factors for power conservation and exploitation in proper dimensions. Therefore, many new routing protocols have been proposed to decrease the power misutilization in the networks.

After several heuristic experiments, evolutionary algorithms have been introduced to effect different scenarios considered. The genetic algorithm, ant colony optimization, fuzzy applications, and firefly algorithms mimic natural phenomena. A genetic algorithm is developed by referring to Darwin’s theory of the existence of the fittest one. The Fuzzy logic optimization technique is a rule-based approach where the fundamental methodologies deal with semantic information. Xin-She Yang introduced the firefly scheme to inspire the blinking of fireflies based on a flashlight to attract other flies for mating or recognizing predators.

With the threshold values T (n) feature, our design has improvised on the energy feature with the DEEC algorithm and DDOS attack detection using the Firefly algorithm, as mentioned below. Sections II and III present various scenarios based on the CH’s clusters and protocol, including LEACH-GA, LEACH with cluster head improvement, and other energy formulations based on threshold values. They are involved in sections IV and V with DEEC-Firefly algorithms that improve the various formulations of T (n), promising the different parametric changes using the linear congestion model for a DDOS attack. This paper is based on the discussed algorithms to conserve energy.

## Literature Review

Chen & Kuo (2019) The attack case *via* TCPIP represents an intriguing problem for the DDOS model to ensure the attack case *via* TCPIP for each set of the packets estimated with the throughput and bandwidth of the network model designed. The authors implement the robust DDOS scenario natural optimization where each mobile node is estimated and predicted using ACO Dong, Abbas & Jain, 2019. The mitigation classification technique utilizes an aspect of reduced/stop packet transfer. An attack in the middle of the way that interrupts the flow of packets is one of the DOS attacks which slows down the data flow and can defend by grouping into various clusters (Abdul Basith & Shankar, 2020a; Abdul Basith & Shankar, 2020b; Spurthy & Shankar, 2020). With network participation of all the nodes for data, transmission needs a large amount of power that can resolve by node optimization. Basha & Shankar, 2021, Yee et al., 2020; Sarkar & Murugan, 2019, Yee et al., 2020.

Energy consumption can manage better by referring to homogeneous fuzzy-K means clustering (Abdul Basith & Dr. Shankar, 2020a; Abdul Basith & Shankar, 2020b Abdul Basith & Shankar, 2021; Seng, Khalid & Yusof, 1999). Classification and clustering improve the energy imbalance in wireless sensor networks (Siva Shankar et al., 2020; Ashokkumar et al., 2021). The wireless networks have several issues in terms of energy used to assemble the heterogeneous mobile Adhoc networks. Fatima, Mahin & Taranum (2019) propose efficient procedures to overcome such challenges. Any disaster must cause damage to the tel-infrastructure. Chen & Liu (2013) suggest a low-cost internet communication system to challenge such a situation (Daanoune & Ballouk, 2020). Presents a novel idea for improving the routing protocol’s energy efficiency. Doncel & Fourneau (2019) emphasize the importance of individual packet energy conservation. Elsmany et al. (2019) have published a comprehensive work on energy-efficient scalable routing algorithms that helps save energy at various levels. The main intention of any energy conservation measure is to enhance the duration of wireless sensor networks by minimizing the energy distortion. Song and FanYanxiang (Yang et al., 2018); A network must refer to the Low Energy Adaptive Clustering Hierarchy (LEACH) protocol for better performance (Singh, 2014, Marappan & Rodrigues, 2016; Al-sodairi & Ouni, 2018; Fan & Song, 2007). We will discuss Energy LEACH and multi-hop LEACH to achieve improved performance. A Genetic Algorithm (GA) is famous as an essential tool for optimizing complicated challenges based on the fitness principle of the gene (Lambora, et al. 2019; Wu et al., 2020). In addition to optimization, it also serves the purpose of machine learning and for research and development. The Firefly algorithm is like swarm optimization and is simple to learn and employ (Avudayappan & Deepa, 2016; Khan et al., 2016).

A cyberattack of this kind is likely to result in significant economic losses for companies and service providers due to the increased operational and financial expenses that will incur (Fatima, Mahin & Taranum, 2019). Machine learning (ML) methods have been more popular in recent years to prevent distributed denial of service (DDoS) attacks. Indeed, with machine learning methods, many defensive systems have been converted into innovative and intelligent systems, which has enabled them to resist DDoS assaults. This article examines current research on DDoS detection techniques that has adapted single and hybrid machine learning methodologies to contemporary networking settings, as well as their limitations. In addition to this, the article covers several DDoS defensive systems that depend on machine learning methods and operate in a virtual environment, such as cloud computing, software-defined networks, and network functions virtualization environments (NFV) (Marappan & Rodrigues, 2016). Because the growth of the Internet of Things (IoT) has received considerable academic interest in recent years, the article also addresses machine learning (ML) methods as security solutions against distributed denial of service (DDoS) attacks in the Internet of Things settings (Avudayappan & Deepa, 2016).

(Gope, Lee & Quek, 2017) The mix of assault methods with different traffic data analyzed has been the most challenging problem in DDoS detection. As part of this article, we introduce Lucid, a practical and lightweight deep learning DDoS detection system that uses the characteristics of convolutional neural networks (CNNs) to categorize traffic flows as either malicious or benign. The four significant contributions are now: (1) a creative framework of a CNN to pinpoint DDoS traffic with limited computational overhead; (2) a dataset-agnostic data preparation method for producing traffic predictions for web security attacks; (3) a stimulation evaluation to illustrate Lucid’s DDoS identification; and (4) an accurate understanding of the alternative model on a resource-constrained computing system (Yee et al., 2020). Lucid can match the current state-of-the-art detection accuracy using the most recent datasets while exhibiting a 40x decrease in computing time compared to the state-of-the-art accuracy rate.

## DDOS Attack and Energy

### ADHOC routing model and DDOS importance

Because of the mobility of nodes in ad-hoc networks, routing has proven to be very difficult. The routes for a given scenario may or may not exist between source and destination. The intermediary nodes’ energy levels must consider when making routing choices in a resource-constrained environment, such as a wireless sensor network (WSN).

In a wireless sensor network, routing methods have been categorized into four techniques: proactive routing protocols [3], reactive routing protocols [4], hybrid routing protocols [5], and location-aware routing strategies. In an aggressive routing scheme, each node keeps its routing table up-to-date by regularly asking its near neighbors for additional routing information. One such system is the Destination Sequenced Distance Vector (DSDV) routing protocol [4], an example of this scheme. Such systems, however, have many significant disadvantages, one of which is the extra cost associated with frequent route changes. On the other hand, reactive routing includes the creation of routes on the fly and will drive by demand. It is basis will implicate the request–response paradigm of communication. Flooding may be used during the initial discovery phase to locate the intended node, and the response phase is responsible for establishing the temporary active routing route. Ad-hoc On-Demand Distance Vector (AODV) Routing and Dynamic Source Routing (DSR) are two examples [5, 6] of such routing techniques.

Many hybrid protocols combine the node-discovery technique of the proactive routing protocol to establish routing paths on the fly to create a hybrid version of the protocol. A hybrid system is the Zone Routing Protocol (ZRP), developed by Cisco [7]. When making routing choices in position-aware routing protocols, the nodes choose the adjacent node that is the most geographically nearest to them. Among the protocols that fall into this category is the Geographical and Energy-Aware Routing (GEAR) protocol. Gear, on the other hand, does not take safety into account. Because asymmetric-key cryptography (RSA-based algorithms) was computationally expensive, most secure methods in WSN relied on symmetric-key cryptography as its foundation.

On the other hand, Symmetric-key cryptography has significant disadvantages in key management, and security depends on pre-shared secret keys. The successful implementation of pairing-based cryptographic algorithms in WSN has opened the door to developing an entirely new platform for deploying asymmetric-key cryptographic methods in WSN. Each node needs the energy to perform any operation such as packet forwarding, receiving defense attacks; however, any node malfunctioning or lacking strength may succumb to a denial of service attack, which can nullify by optimizing the nodes as the firefly algorithms.

**Figure 1:**
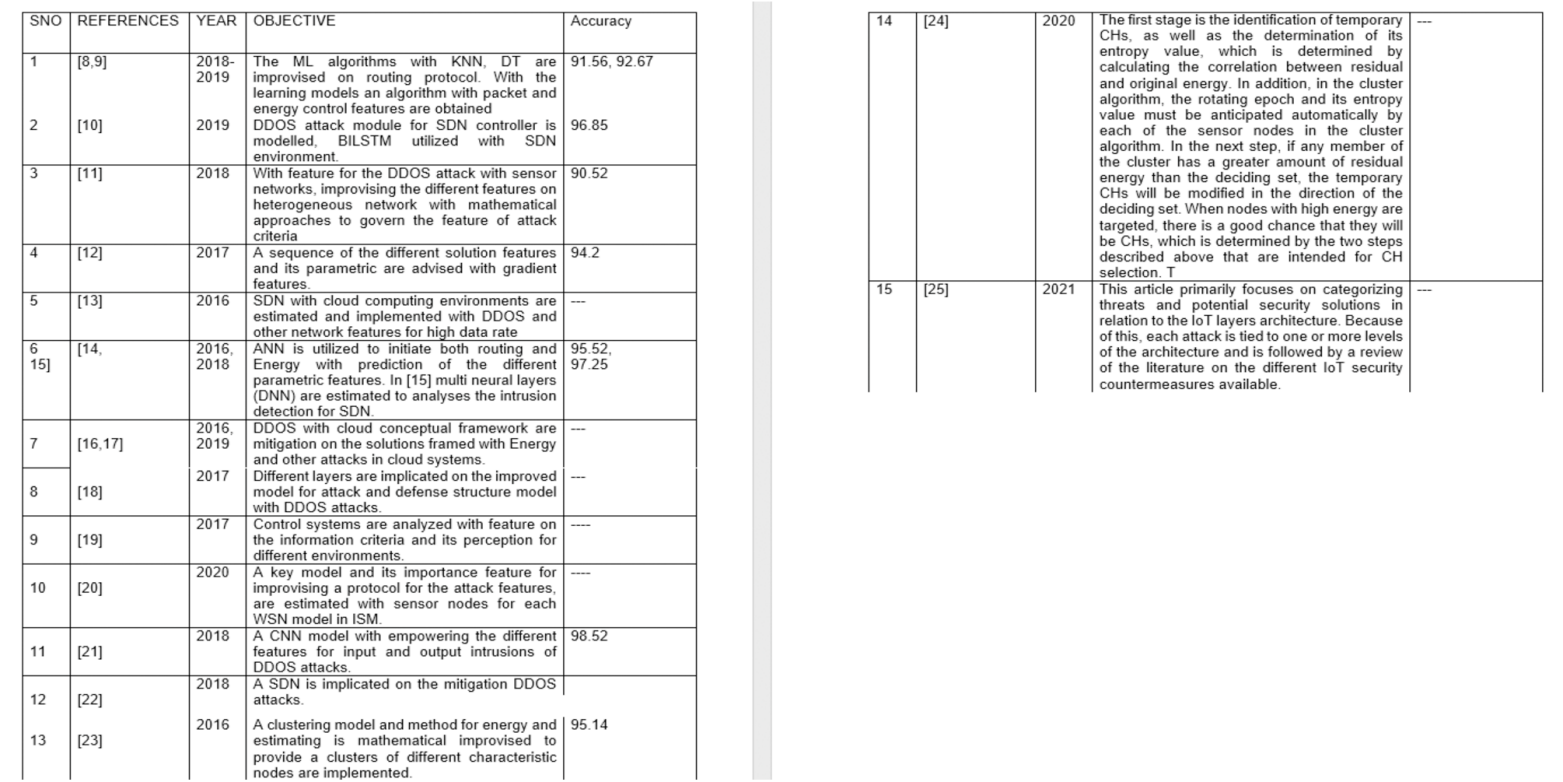
Representing the literature survey of the DDOS AND CRA attacks.

The features of the different algorithms and their analysis by other authors have been analyzed. Figure 1 and Table 1 are the same, but only a clearer perspective has been mentioned using figures and table formatting.

**Table 1:** (Razzaque & Dobson, 2014–Singh, 2014) Representing the literature review on DDOS-CRA attacks.

SNO	References	Year	Objective	Accuracy
1	Siva Shankar et al., (2020), Hassanat et al. (2016)	2018–2019	The ML algorithms with KNN, DT are improvised on routing protocol. With the learning models an algorithm with packet and energy control features are obtained	91.56, 92.67
2	Chen & Kuo (2019)	2019	DDOS attack module for SDN controller is modelled, BILSTM utilized with SDN environment.	96.85
3	Toumia & Hassine (2021)	2018	With feature for the DDOS attack with sensor networks, improvising the different features on heterogeneous network with mathematical approaches to govern the feature of attack criteria	90.52
4	Doncel (2021)	2017	A sequence of the different solution features and its parametric are advised with gradient features.	94.2
5	Abdul Basith & Shankar (2020a)	2016	SDN with cloud computing environments are estimated and implemented with DDOS and other network features for high data rate	–
6	Abdul Basith & Shankar (2021), Abdul Basith & Shankar (2020b)	2016, 2018	ANN is utilized to initiate both routing and Energy with prediction of the different parametric features. In [15] multi neural layers (DNN) are estimated to analyses the intrusion detection for SDN.	95.52, 97.25
7	Spurthy & Shankar, 2020, Basha & Shankar (2021)	2016, 2019	DDOS with cloud conceptual framework are mitigation on the solutions framed with Energy and other attacks in cloud systems.	–
8	Ashfaq, Ali Safdar & Ur-Rehman (2018)	2017	Different layers are implicated on the improved model for attack and defense structure model with DDOS attacks.	–
9	Fatima, Mahin & Taranum (2019)	2017	Control systems are analyzed with feature on the information criteria and its perception for different environments.	–
10	Marappan & Rodrigues (2016)	2020	A key model and its importance feature for improvising a protocol for the attack features, are estimated with sensor nodes for each WSN model in ISM.	–
11	Avudayappan & Deepa (2016)	2018	A CNN model with empowering the different features for input and output intrusions of DDOS attacks.	98.52
12	Gope, Lee & Quek (2017)	2018	A SDN is implicated on the mitigation DDOS attacks.	
13	Yee et al. (2020)	2016	A clustering model and method for energy and estimating is mathematical improvised to provide a clusters of different characteristic nodes are implemented.	95.14
14	Razzaque & Dobson (2014)	2020	The first stage is the identification of temporary CHs, as well as the determination of its entropy value, which is determined by calculating the correlation between residual and original energy. In addition, in the cluster algorithm, the rotating epoch and its entropy value must be anticipated automatically by each of the sensor nodes in the cluster algorithm. In the next step, if any member of the cluster has a greater amount of residual energy than the deciding set, the temporary CHs will be modified in the direction of the deciding set. When nodes with high energy are targeted, there is a good chance that they will be CHs, which is determined by the two steps described above that are intended for CH selection. T	–
15	Singh (2014)	2021	This article primarily focuses on categorizing threats and potential security solutions in relation to the IoT layers architecture. Because of this, each attack is tied to one or more levels of the architecture and is followed by a review of the literature on the different IoT security countermeasures available.	–

## Leach A-B implementation

Heinzelman, Chandrakasan & Balakrishnan (2002) Introduce the LEACH, Low-Energy Adaptive Clustering Hierarchy, hierarchy model term, the first and most prominent case of the WSN energy reduction scheme. This protocol transmits the various assumptions on power transmission and its associated formulation model to the base station BS with precise transmit power. The node ensures the control capability for each set of conditions is adjusted to withhold the Tx-power to address the power computation observed while providing the MAC protocols to ensure the signal processing functions are modeled. LEACH protocol improvises with timing scenarios on states of clusters such as setup, steady-state, framing, and rounding the data to ensure the generation of a practical solution after passing through the threshold.

T is the sensor nodes considered with different values where the cluster head becomes the futuristic cluster current round if the threshold number is chosen. It iterates until Eq. (1) is satisfied.

(1) \begin{eqnarray*}T \left( n \right) = \left\{ \begin{array}{@{}l@{}} \displaystyle \frac{p}{1-p \left( rmod \left( 1/p \right) \right) } n\in G \\ \displaystyle 0 others \end{array} \right\} .\end{eqnarray*}

Here, T(n) represents the overall sensor nodes probability, p defines the probability for which one node is active. N represents number of nodes and G represents the overall cluster.

The LEACH represents an advanced protocol where each energy set is remodelled with a heterogeneous probability of each node on failure and alive scenario. The synchronization model on clocking applies with a factor of each node observed on every round where the maximum energy is estimated with selected clusters head which could provide the path on each set of distances observed from the base station. Similarly, a balanced LEACH protocol is considered, which utilizes the residual energy equation established for each sensor node. The decentralized approach provides the position of source and destination, which improvises three cases on selecting CHs, cluster formation, and data transmission information accessed with multiple scenarios. Hence, the current node’s energy dissipation for each CH’s destination estimate is established on recent rounds.

## a. LEACH with genetic algorithm.

One such feature on WSN’s has been proven a newer feature for reducing energy on sensor nodes and implicating higher data transfer with a Nature-based algorithm as a Genetic algorithm. Data groups and their functional values have become a critical scenario for using genetic algorithms in wireless sensor networks. They allow the development of the most energy-efficient and stable clusters. GA is used exclusively for integrated grouping measurements in more spectacular execution hubs such as the BS. Generally, a high-quality gene in the chromosomes communicates with a sensor center. The duration is defined when the network model has developed the characteristics of the architecture parametric parameters, ensuring the various sensor hubs that interact either individually or in clusters.

LEACH-GA improvises the hierarchy of the different clusters governed by the features on the nodes identified as the characteristic nodes. These are estimated with the specific models to be analyzed in the different iterative scenarios that govern the energy or path features—the value of T(n) implicating the probability analysis on each set of sensor node creation. With the propositions mentioned per the algorithm, a model estimation of the different scenarios for each design stage is made.

## b. DEEC-FIREFLY.

In DEEC-FIREFLY, the most effective method of increasing the endurance of WSN is to combine WSN with tremendous energy, which is referred to as the Head of the group, also known as the Cluster Head (CH). CH becomes reliant on other clusters for intra-cluster and inter-cluster communication. The energy level of CH increases the longevity of a set in a fully functional WSN. The difficult task is determining the amount of energy used by heterogeneous networks while simultaneously developing the clustering method. The proposed work is titled ”Novel Distributed Entropy Energy-Efficient Clustering Algorithm,” or short for ”DEEEC for High-Speed Networks (HWSNs), and it is based on the Firefly Algorithm CH (DEEC FA-CH) Selection. The DEEEC Algorithm, represented by the letter CH, is divided into two phases. The identification of temporary CHs and their entropy value, which is determined using the correlative measure of residual and original energy, is performed in the first stage. In addition, each sensor node must automatically anticipate the rotating epoch and its entropy value in the clustering algorithm. Next, if any cluster member has considerable residual energy, the deciding set where the particular nodes representing the cluster heads are minimized in the sensor node’s direction is active. When nodes with high energy are targeted, there is a good chance they will be CHs, which is determined by the two steps described above intended for CH selection. Simulating the DEEEC algorithm requires the use of MATLAB software. Compared to existing conventional clustering protocols, which are utilized in heterogeneous WSNs, the simulated results of the proposed DEEEC Algorithm provide favorable outcomes in terms of energy consumption and improved lifespan.

A sensor node is appropriately installed in most geographical locations where energy solutional values become minimum. The extraction of wide-ranging network architectures for simulation is accomplished by assuming random node positions as realistic as possible. A fixed sensor network is established after the deployment of the sensors, and data is typically sent to a stationary base station, which is situated at a distance from the sensing part. In contrast to DEEEC, if the middle region is located as the source point, the assumption is made to ensure that the BS is correctly positioned in the organization (0, H).

Each node is acquainted with the overall energy used in the network by retrieving a data packet from the BS. When considering the whole HWSN, the equation represents the total energy.

(2) \begin{eqnarray*}{E}_{ntotal}=\sum _{i=1}^{N} \left( 1+{a}_{i} \right) {E}_{n0}={E}_{n0} \left( N+A \right) \end{eqnarray*}

Here, *a*_*i*_ represents random number varying values from (0-1), m being the fraction of advanced nodes of the total nodes N also A refers the different lower bound energies available. The multipath-channel form is utilized in various situations depending on the location between the transmission and reception. The amount of energy required to broadcast an l-bit packet over a long distance is referred to as

(3) \begin{eqnarray*}{E}_{tx}=l{E}_{ntotal}+{d}_{min}^{2}l{e}_{fs}+{d}_{min}^{4}l{e}_{fs}\end{eqnarray*}

From Eq. (3) *E*_*tx*_ representing the transmitted energy, l represents the data bits to the sender, ${d}_{min}^{2}$ minimum distance squared value from selected node to Tx and Rx, *e*_*fs*_ being the estimated amplified energy

Similarly for receiving side,

(4) \begin{eqnarray*}{E}_{rx}=l{E}_{ntotal}\end{eqnarray*}

Finally for all the iterations,

(5) \begin{eqnarray*}{E}_{t}={E}_{rx}+{E}_{tx}\end{eqnarray*}

With Eq. (4) our design improvises the design feature to evaluate it,

(6) \begin{eqnarray*}di{s}_{min}=W/\sqrt{2}\pi e{x}_{c}\end{eqnarray*}

With the estimation on Eq. (5), the minimum values for the distance are statistically modeled with *ex*_*c*_ and W.

W with the probability observed as the weights. The simulation parametric are mentioned in the simulation results section.

## Fuzzy logic for leach protocol

The design factor of the blocks considers three parameters. Parametric factors such as distance, velocity, and density in the communication model implicate the different solutions of the energy model. Karimulla Basha and T. N. Shankar, 2021. The fitness values are acquired with fuzzify-defuzzify on the required data considered. A characteristic map is created with conditional changes in parameters for each fitness value observed from defuzzification.

**Figure 2:**
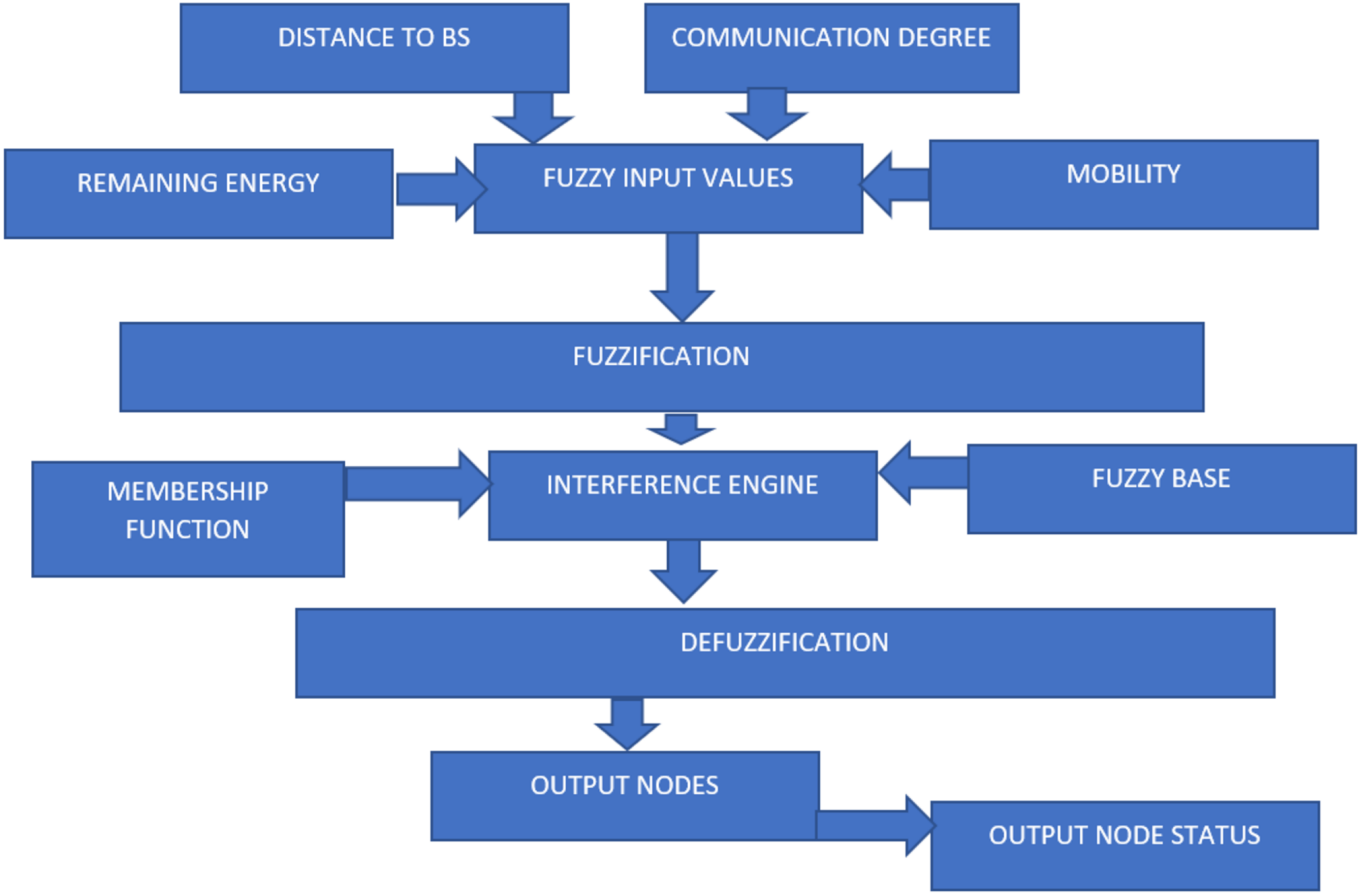
Representing the flow diagram of fuzzy logic optimization.

Figure 2 depicts the structure of the fuzzy system in various requirement scenarios where each set of the cluster justifies the importance of the implementation of WSN features for each specific value. The energy and network lifetime models use the prediction values with the different case studies.

## Genetic algorithm.

Some potential solutions create a dilemma with the population/pool of possible solutions. The solving techniques process experience recombination and replication (as in natural genes), lead to the resolution and eventually pass on as new life-forms. The technique is used and handed down from generation to generation. Individuals (who have better fitness) are granted a higher priority because the preferred option (or product) has the advantage of being made and given to healthy individuals (the best-fit ones are assigned a high priority), consistent with the ”survival of the fittest” theory of Charles Darwin.

Instead of discarding each age and going back to the previous one, progressing towards better individuals or alternatives is quite impossible before hitting a hard limit. Assume at random to do much better than just a simple search by gathering knowledge from the past (which depends on using the “try all and pick out the strongest” algorithm) for the combinatorial experience.

## Genetic algorithm phases.

The criteria on the current algorithms for the design features improvise in different scenarios with each set of parametric to ensure the correct and predicted outcome where the section of modeling establishes as the problem-solution that depends on various states.

*Population*: Solution set on each subset of problems assigned to specific criteria on specifications such as node estimation and characterizing the node value states.

*Chromosomes*: A single solution set on each set of iterated outcomes observed on a single phase.

*Gene*: Elemental values on each position for the variation observed when a chromosome generates.

*Genotype*: Computation model for each set of problem solutions.

*Encoding and Decoding*: Spatial change on variables and the positional values.

**Algorithm 1**

*Begin*

*Ch*_*i*_ ← Encode the chromosomes.

 do While

*F*_i_ ← O(Ch_i_) // Use the fitness function to learn that is fittest or not.

*C*_*i*_ ← Ch_i_ Crossover Ch_i+1_

*M*_i_ ← M(C _i_) // M is the mutation

*Ch*_*i*+1_ ← *F(M*_i_*)*

End do

End

## WSN with Genetic Algorithm

GA is used in several domains, including wireless sensor networks (WSNs)—including planning, data transmission, directing the different signal data according to an existing model, and bunching various collections of energy-efficient clusters. Data groups and their functional values can be used in genetic algorithms in wireless sensor networks as they allow the development of the most energy-efficient and stable sets. GA can exclusively utilize it for integrated grouping measurements in spectacular execution hubs like the BS. Generally, a high-quality gene in the chromosomes communicates with a sensor center. The duration defined when the network model is under development reflects the characteristics of the architecture parametric to ensure the various sensor hubs that interact either individually or in clusters.

***Algorithm 2***

*Cluster(Cl)* ←*Population*

*Begin*

*Ch*_*i*_ ← Encode is one of the features of a cluster node as a chromosome.

 do While

*F*_i_ ← *O* (*C*_*i*_) //Get the fitness genes by the optimization function.

If (*F*_i_ ——fittest)

*C*_*i*_ ← Ch_i_ Crossover Ch _i+1_

M_i_ ← M(*C*_*i*_) // M is the mutation

*Cl*_i+1_ ← *F(M*_*i*_*) // Form a new cluster with fittest nodes*

End do

End

### Proposed model

The proposed model refers to the link state modeling with the threshold algorithms that ensure the different sets of sensor nodes that are active and passive values with each group of MPRs chosen. This formation happens by utilizing Dijkstra’s algorithm by considering various sensors and their relative positions. This design improvises the model with a firefly algorithm, ensuring the best swarm for the energy optimization and packet (alive and dead clusters also). The proposed prototype imparts the design features with firefly optimization to produce a better outcome for each set of parameters such as energy, network lifetime, and packet drops in alive and dead scenarios. The criteria of each parameter can be observed and estimated for each functional model. Our design uses a specific optimization algorithm as a firefly for the Linear Congestion Model (LCM) with different parametric features. Considering elements in the linear congestion model can utilize the entropy and gain values from each node and its selected MPR’s.

### Problem statement

a. Analyze the current design optimizers with energy equations using the firefly scheme with the best performance features.
b. The proposed design implicates the factors of the link state with the firefly algorithm to provide the root-sigmoid calculations over the selected nodes.
c. Reduction on functional parameters: Energy, transmission range, packet rate, load on a network must emphasize the (5).

***Algorithm 3***

Number of fireflies = *m*

*Begin*

*B* ← Brightness

*j* ←1 to *m*-1

*k* ←*j* +1 to *m*

For Loop

if (*B*_k_ >*B*_j_)

Print “Fly firefly j to k.”

end for

End

***Algorithm 4***

Number of nodes in WSN = m

Begin

*S* ← Energy level

*j* ←1 to *m*-1

*k* ←*j* +1 to m

For Loop

if (*S*_k_ >*S*_j_)

Print “Select the path *j* to *k*.”

end for

End

### Formulation for energy-efficient

From the perspective of design scenario on LEACH-FA, With Fuzzy logics on Energy values which are governed with sigmoid function mentioned below:

(7) \begin{eqnarray*}S \left( i \right) =\sum _{\mathbi{i}=1}^{\mathbi{N}}1/(\sqrt{(1+{e}^{-i})})\end{eqnarray*}

The functionality of the S represents the design solution of the nodes that appear at the given timing aspect, where each set of the design is parametrically considered with active and dead cells from the equation.

(8) \begin{eqnarray*}\mathbi{F} \left( \mathbi{i}\gt k \right) =\sum _{\mathbi{i}=1}^{\mathbi{N}}({\mathbi{n}}_{\mathbi{ i}}\ast MPr \left( \mathbi{i} \right) +\sigma \ast S(\mathbi{i}))\end{eqnarray*}

F represents the solution model where all the active and alive nodes in a cell region are established with Eq. (8), *and n*_i_ stands for the involved nodes**. Σ** is the best prediction of firefly optimization for all the iterations.

(9) \begin{eqnarray*}{\mathbi{PE}}_{\mathbi{MPRs}}=\mathbi{F}(\mathbi{i}\gt k)\end{eqnarray*}

(10) \begin{eqnarray*}{\mathbi{PE}}_{\mathbi{Head}\text{_}\mathbi{cluster}}=\gamma \ast {\mathbi{W}}_{\mathbi{i}}\ast {\mathbi{D}}_{\mathbi{min}}+\mu MPr \left( \mathbi{i} \right) +\mathbi{E}(\mathbi{i})\end{eqnarray*}

Here $E \left( i \right) $ represents the entropy of each selected MPR selected.

Hence the total Network energy estimated is:

(11) \begin{eqnarray*}\mathbi{P}{\mathbi{E}}_{\mathbi{T}}={\mathbi{PE}}_{\mathbi{Head}\text{_}\mathbi{cluster}}+{\mathbi{PE}}_{\mathbi{cluster}\text{_}\mathbi{MPRs}}\end{eqnarray*}

Here *γ*, *μ*, *σ*,  are estimated probabilities for the optimized values of the best solution in the modified firefly algorithm based on the energy equations on active and dead scenarios. Power observed based on the algorithm (LS_MFT) on the different MPR active nodes and control with distributed energy model implementation with an ensemble approach.

### Rate control formulations

The actual rate *ACR*_*g*_ is given by

(12) \begin{eqnarray*}AC{R}_{ij}=W\ast A{R}_{ij}\end{eqnarray*}

Where,

(13) \begin{eqnarray*}W= \left[ \frac{Lc}{\sum A{R}_{ij}} \right] \end{eqnarray*}

If the source receives the congestion bit (CB), the allocated rate

(14) \begin{eqnarray*}A{R}_{ij}=AC{R}_{ij}-\delta \end{eqnarray*}

The rate monitoring function measures the traffic rate of a given in-out stream over a time interval T.

(15) \begin{eqnarray*}M{R}_{ij}={C}_{ij}/T\end{eqnarray*}

If the estimated intensity MR_ij_ is greater than the real rate ACR_ij_ for the following time frame, the flow is classified as an assault value. The state of the request is set to REJECT, and the FMM records the corresponding source of IP address.

### Firefly optimization:

Algorithm improved:

1. Improve the position vector by the formulation based on the energy equation F(I > k) as:
  a. (16) \begin{eqnarray*}{x}^{k+1}={x}^{k}+{e}^{-{r}^{2}\omega } \left( {x}_{i+1}^{k}-{ \frac{{x}_{i}^{k}}{\sqrt[4]{1+{e}^{-x}}} }^{2} \right) +\delta \ast xmi{n}_{i}^{k}\end{eqnarray*}
  b. *Here x is the expected outcome for each energy−optimized value observed for Network model*
  c. **ω* and *δ* are the step factors, xmin are feature values varies from (0,1).*
  d. *e*^−*r*^2^*ω*^ *is a characteristic feature for Expected value for the Energy optimization with Minimal step variance of 0.785 value.*
2. Secondly, update Eqs. (7) and (16) for each set of values observed for energy implementation using Link state and DEEC.
3. Thirdly implement this optimization using energy and active and passive nodes.
4. Finally, update the iterations, ensuring the correct and accurate results have been observed.

### Link state firefly optimization

1. Initialize the design with a flag and graph where each node utilizes the maximum value.
2. Estimation on each tree graph can generate from the selection of different MPRs based on the Dijkstra technique to calculate the optimum with the minimum distance between the nodes estimated for MPR’s.
3. Optimization of the threshold values for each selected MPR’s generated from Eq. (1).
4. Hoping on iterations changes would suffice the selection of MPR’s onto minimum energy model applies for given criteria.

### Link state model for multi-point relays in wsn

**Figure 3:**
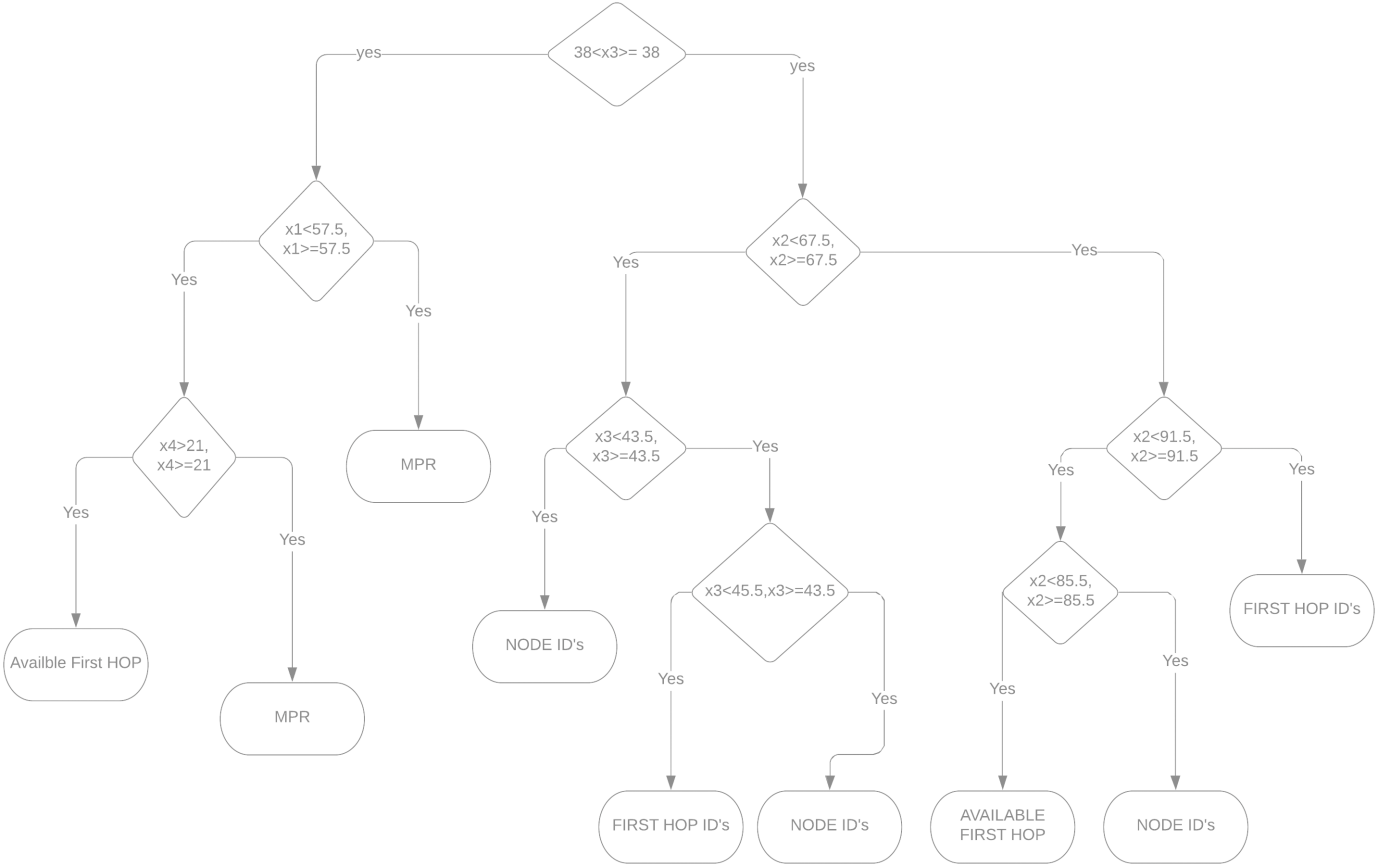
Represents hoping, node-ids, MPR’s branched with the decision tree algorithm.

The current link state design incorporates a switching node to forward packets labeled with routers. Figure 3 shows two scenarios for learning models: design accuracy and energy reduction capabilities. With the formulation provided on Eqs. (2)–(3), our design implicates the different distance measurement from each set of cluster nodes.

### Link state prediction with energy clustering

According to the prediction study of the new hybrid protocol, various relay points emerge in response to the threshold and total node considerations. To start the multi-point segmentation with defined hops, created flags and exit flag conditions must be updated and current authentication dedicated to the cluster nodes associated with the MPR’s. Initial Activation and its values for each active node can represent either alive or dead scenarios for each cluster accumulation on the current network using the cluster head selection.

### The formulation for defensive mechanism for energy model and resource consumption

The behavioral model has two distinct phases: (i) query and transfer and (ii) bandwidth request and reply controls. The source IP address, destination IP address, port, and flow ID is essential for making a query on a node in a request packet. The proposed system computes the FMT data along a vector. If the intermediate node receives a return answer on the back-to-back route, that modifies the FMT and pushes forward into the return path by transmitting the BnBW value to the next node. For the initial determination of ABW, the available bandwidth is tested. If the bandwidth is accessible, this excess can provide the flow, ABW > BnBW. Otherwise, BnBnB is reduced to AB. In this case, the reply packet’s assigned rate is now RRij. An FMM entry is created if the stream before j was previously inactive. Following the REPLY packet, the message is sent in the same direction. The source discards the real-time flow based on the BnBW value at this stage.

### Proposed defense algorithm

With the parametric feature of the design modules based on the DEEC-FIREFLY, our design has initiated different hybrid states for the IFFLY (Improved Firefly). The node features on the particular time and simulations for the sender, and receiver id depends on the minimum energy values observed with the LSF algorithm. The proposed algorithm (IFFLY and LSF) would operate with different features based on standard and attack modules.

**A. Algorithm:**

1. Initiate a timing scenario with a start and stop values for the design set.

2. With Link state firefly optimization, the design with the estimation of the minimum values for different nodes generated a threshold based on the Th_min (ensuring the minimum distance from each node observed).

3. Finally, we check whether the Links are optimized with Th_min and its corresponding nodes with E_min.

**B. ATTACK MODULE:**

DDOS attacks are consistent with not having users access the correct information with available resources. The initiating of the attack feature would represent the packet that is not enabled for the attacker node from the sending to receiving to the neighboring nodes. With the characteristic of the Link states, we have established that selected MPR’s with the least distances are entitled to features of Firefly optimization based on the DEEC protocol for parametric network features. With the timing feature, we provide a condition that governs the design as attack and defense mechanism as Th_min and E_min are less than that T_r*E_min, T_r*Th_min. T_r is the estimated probability for observing alive and dead nodes on the Routing protocol as the Links state mechanism.

**C. Algorithm:**

1. Create a Timing event for any Node as attack node when Th_min <Th_min*T_r

2. The attack rate of 0.49 for Th_min, Emin is 0.9*E_min

3. Scanning and analysis for each set of the design model and its attack and defense mechanism features are established for every iteration.

4. 1,000, 5,000, and 10,000 iterations are utilized to estimate the thresholds and Energy minimization.

## Results and Discussion

**Figure 4:**
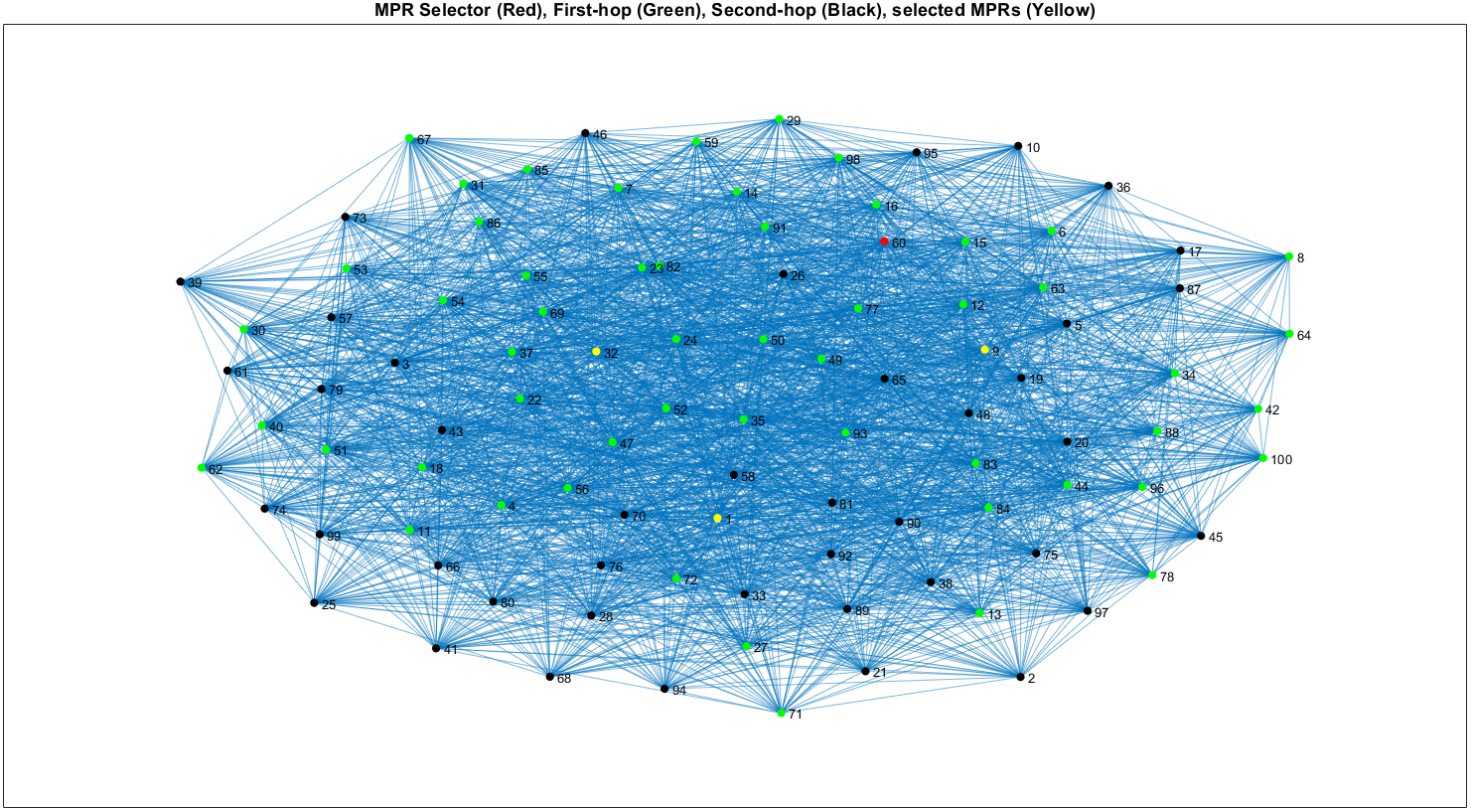
Representing WSN structure on LS-DT algorithm model for MPR selection.

Figure 4 covers the orthogonal least squares (OLS) algorithm for providing the different selections of MPR as mentioned. Also, it describes the condition of the network model for which each set of the design criteria on the active and passive nodes are available in a short range refers to the orthogonal least squares OLS algorithm. The connection of 47 nodes with the multi-point relay MPR selection indicates the green color and MPRs by the yellow.

*Ls-Dt Defines Link State Decision Tree, MPR Defines Multi-Point Relays, Ols Defines Orthogonal Least Squares, DEEC Defines Deterministic Energy Efficient Clustering Protocol, Leach-Fa Defines Leach-Fa Defines Leach-Fa Defines Leach-Fa Defines Leach-Fa Defines Leach-Fa*

**Figure 5:**
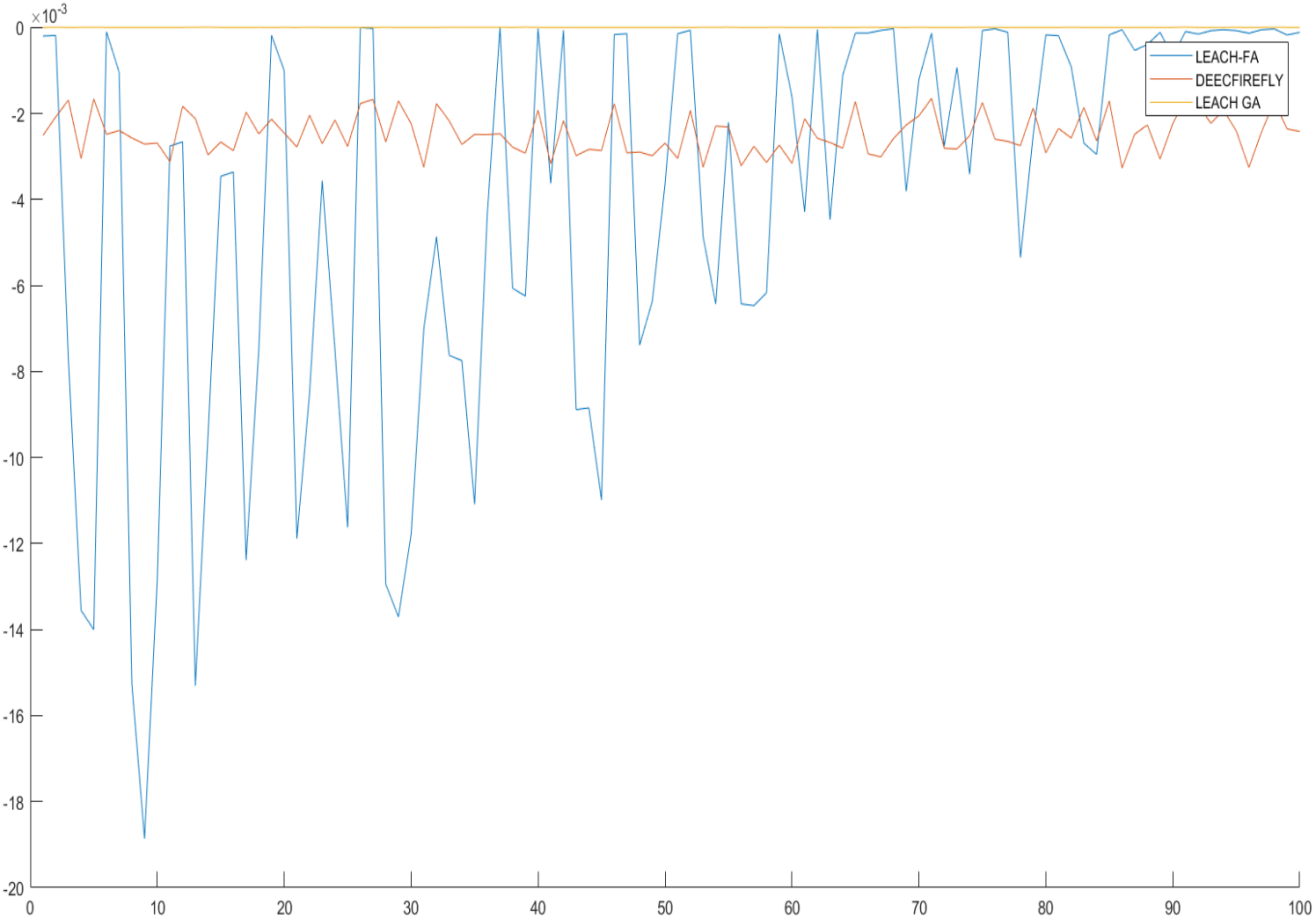
Representing the minimum energy values for the MPR-node at each iteration length of 5000.

The graph provides the least energy simulation values, as mentioned in Fig. 5, for 5,000 iterations of the design matrix 100X100. The experiment was conducted with various values of the OLS algorithms and provided a different scenario of the energy reduction from the protocol utilization. The multi-point relay (MPR) of the iterations 93, 54, and 1 ensures the sets of changes in each iteration’s minimum and maximum values.

**Figure 6:**
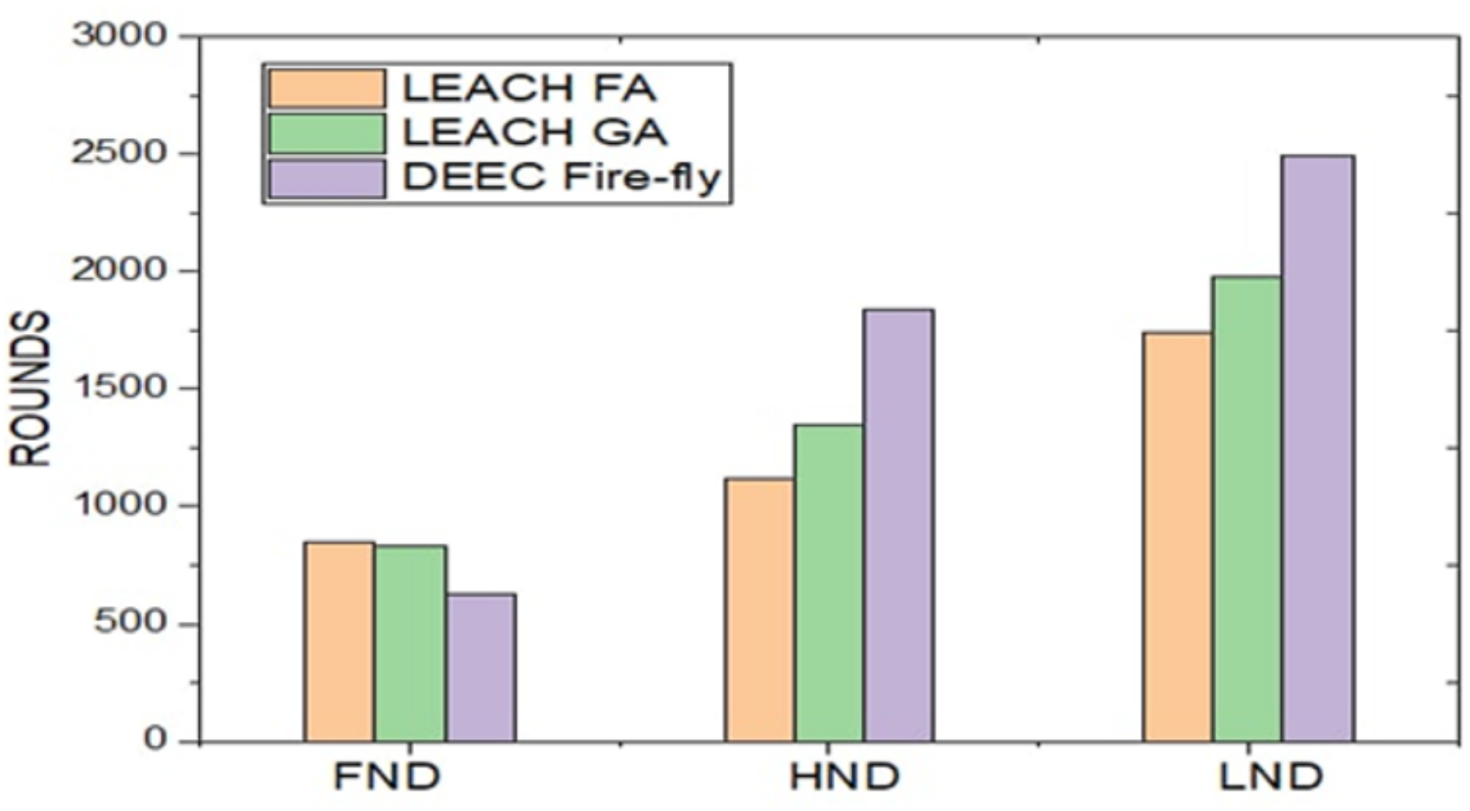
Representing the minimum energy clustering for all the three different algorithms implemented.

Figure 6 demonstrates the output of simulations for the proposed scheme with the LEACH fuzzy application (FA), Genetic Algorithm (GA), and DEEC Fire-fly scheme. The above bar graph describes the first node die (FND) at the 830th iteration for fuzzy application (FA), the 800th for GA, and 520 of the proposed one. Half-node dies (HND) measurement is 1200 for FA, 1500 for GA, and 1820 for the project. Finally, the last node die (LND) calculation is 1,600 iterations of LEACH FA, 1820 for GA, and 2490 for the suggested algorithm.

**Figure 7:**
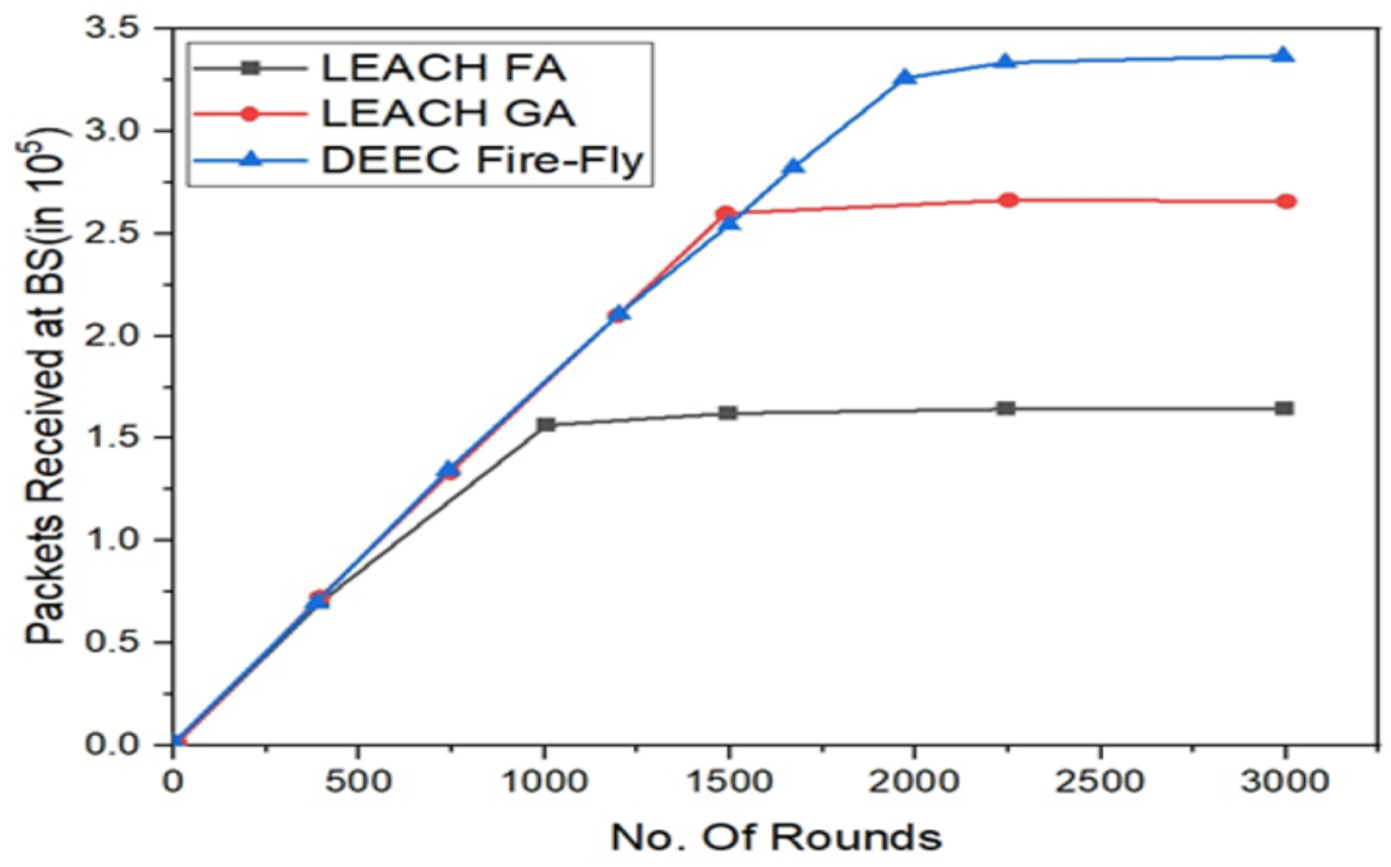
Representing the active nodes observed for all the three different algorithms implemented.

The number of data packets received by the base station is also a parameter for evaluating the high energy efficiency of the utilization rate. The more balanced the energy distribution in the network, the more packets the base station can receive Fig. 7 observes that the number of packets received at the BS for LEACH, LEACH-C, SEP, and

LEACH-VA protocols, where the length of one packet is 4000 bits. As can be seen from this picture, LEACH-VA Increases packet counts received at the BS by 71.4% when compared to LEACH, 33.3% when compared to LEACH-C, and 14.3% when compared to LEACH-C. Against SEP. The significant increase in packet counts received by the base station reduces the probability of cluster head clusters. It effectively reduces negotiated communication consumption within the group of sets. Based on the stable number of cluster heads and the geometric principle of the Voronoi diagram, the clusters are more uniform, the energy consumption between the sets is better, and the energy utilization of the unit nodes is also improved. Moreover, in the paper, a multi-hop transmission routing protocol according to an ant colony optimization algorithm is used to forward the data packets of a long-distance cluster head by a neighboring cluster head of the BS to reduce the energy consumption of direct communication.

**Figure 8:**
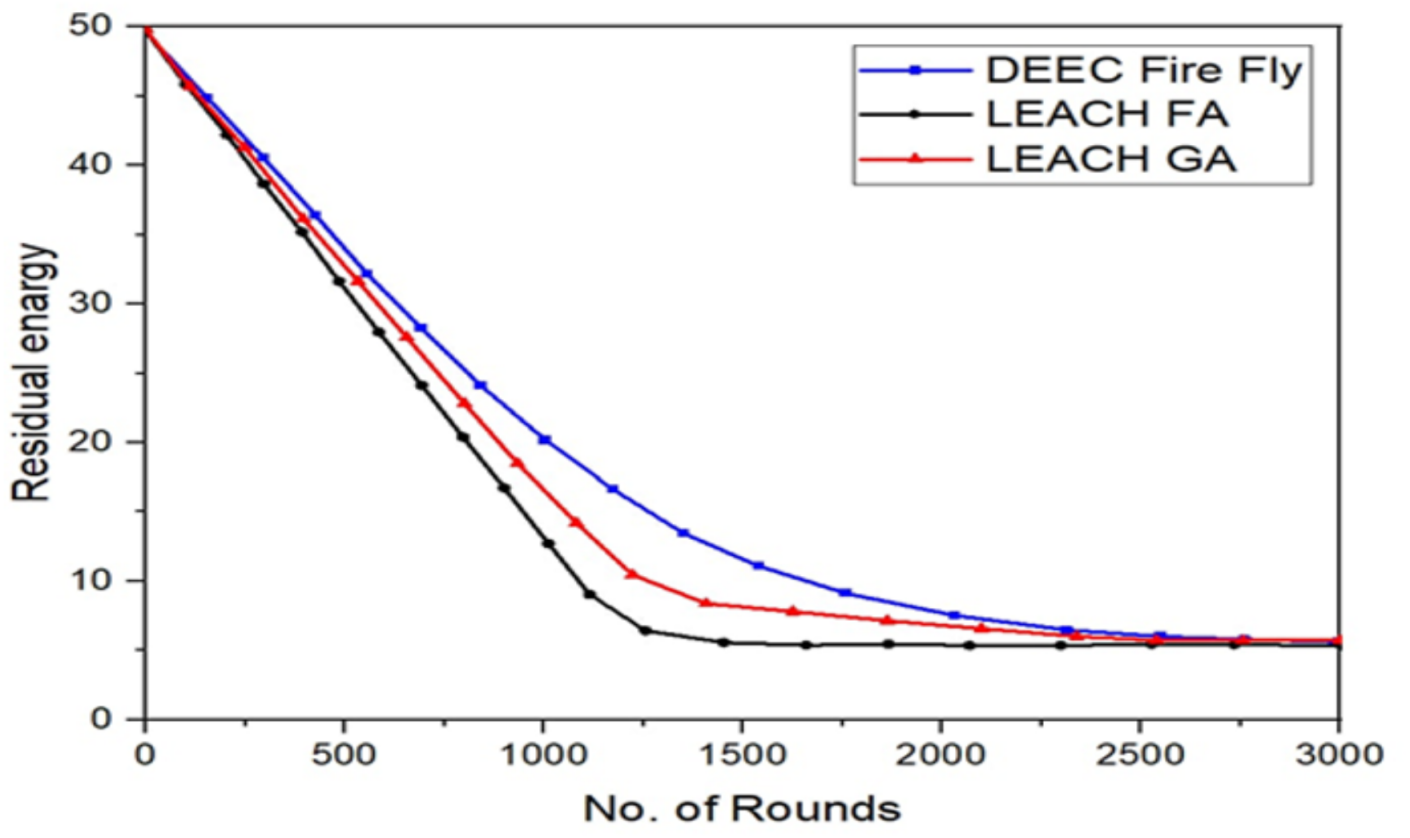
Total remaining energy of nodes in LEACH and the proposed protocol.

Figure 8 compares the proposed solution to LEACH according to the remaining energy metric.

The remaining energy is the difference between the initial and consumed energy. From these curves, we can see that the remaining energy of total sensor nodes in the proposed protocol gradually decreases. Compared to the classical LEACH approach. Then, at around 1200, it is almost null in the LEACH, while in the proposed protocol, it is still more than 15% of the initial energy of all the network nodes. From these results, we can see that the proposed protocol in this paper can optimize energy consumption and extend the stability of the network better than the original LEACH protocol.

Tabulations:

From this perspective on Table 1, our design feature implicates the different sets of the survey publications as references and its accuracy parametric as mentioned in Table 1. References Abdul Basith & Shankar (2021), Abdul Basith & Shankar (2020b) and Avudayappan & Deepa (2016) provide the highest precision as per the design criteria are noted with designs that did not specifically mention the design accuracy.

From Figs. 5–7 in Table 2, we have represented a practical solution that utilized the design importance based on the metric equations proposed for firefly optimization and its essential feature for energy and distance reduction based on the formulation model of the design scenario. This model provides extensive usage in WSN as the observed minimum energy would be around 85-150 dB as a minimum as possible for iterations 1,000, 5,000, and 10,000. These scenarios are observed even on alive and dead nodes, modeled based on the firefly equation, ensuring the correct optimized values.

**Table 2:** Representing the different Algorithms and its Energy minimazation values comparisons.

Feature	Total iterations	Existing algorithm models		Proposed algorithm
Energy	Total iterations	Leech	DEEC firefly	HSLP algorithm
Minimum energy observation for 1k iterations	1,000	−45.42 dB	75.19 dB	85.89 dB
Minimum energy observation for 5k iterations	5,000	−89.34 dB	−90.75 dB	−119.34 dB
Minimum energy observation for 10k iterations	10,000	−101.23 DB	−105.23 dB	−147.23 dB

## Conclusion

Saving energy is challenging within limited resources for packet forwarding in a wireless network. So many methods have been proposed for node optimization to eliminate the number of forwarding points and save resources. The Firefly algorithm is one of the best schemes for improving efficiency in LEACH as an alternative to randomly choosing the number of cluster heads, which consumes more power for packet dissemination in such an environment. As the output of simulations demonstrates through various graphical representations, the proposed scheme can be stabilized based on the power level and make the network more efficient based on the power level. In the future, the design features are implicated with the enhanced-LEACH protocols to learn the story of energy conservation and the duration of the system lifetime. There is an advantage over traditional network equipment in a resource-constrained setting, though this is only true about specific types of technology that can operate with WSN. As mentioned in the literature survey, the security of nodes is represented with different algorithms but may put the network at risk. The defend-but-do-not-abuse (Do Not Distrust) protocol can refer to trust relationships with WSNs. Since a wireless sensor network must respond quickly, its speed must be low. Additional research is needed on current wireless channels and applications and enhancing the trust framework itself. Thus, we will use risk evaluation, recycling, and novel approaches that combine trust and energy efficiency to reduce risk.

## Supplemental Information

10.7717/peerj-cs.845/supp-1 Supplemental Information 1 Hybrid distance Code

10.7717/peerj-cs.845/supp-2 Supplemental Information 2 Final solution for DDOS attack coding

## Additional Information and Declarations

### Data Availability

The following information was supplied regarding data availability: The codes of the design are available in the Supplemental Files.
